# Immune Cell Contribution to Mammary Gland Development

**DOI:** 10.1007/s10911-024-09568-y

**Published:** 2024-08-23

**Authors:** Ramiah Vickers, Weston Porter

**Affiliations:** Department of Veterinary Physiology and Pharmacology, College of Veterinary Medicine, Texas A&M University, College Station, TX 77843 USA

**Keywords:** Mammary gland development, Innate immunity, Adaptive immunity, Pregnancy, Lactation, Postpartum breast cancer, Inflammation

## Abstract

Postpartum breast cancer (PPBC) is a unique subset of breast cancer, accounting for nearly half of the women diagnosed during their postpartum years. Mammary gland involution is widely regarded as being a key orchestrator in the initiation and progression of PPBC due to its unique wound-healing inflammatory signature. Here, we provide dialogue suggestive that lactation may also facilitate neoplastic development as a result of sterile inflammation. Immune cells are involved in all stages of postnatal mammary development. It has been proposed that the functions of these immune cells are partially directed by mammary epithelial cells (MECs) and the cytokines they produce. This suggests that a more niche area of exploration aimed at assessing activation of innate immune pathways within MECs could provide insight into immune cell contributions to the developing mammary gland. Immune cell contribution to pubertal development and mammary gland involution has been extensively studied; however, investigations into pregnancy and lactation remain limited. During pregnancy, the mammary gland undergoes dramatic expansion to prepare for lactation. As a result, MECs are susceptible to replicative stress. During lactation, mitochondria are pushed to capacity to fulfill the high energetic demands of producing milk. This replicative and metabolic stress, if unresolved, can elicit activation of innate immune pathways within differentiating MECs. In this review, we broadly discuss postnatal mammary development and current knowledge of immune cell contribution to each developmental stage, while also emphasizing a more unique area of study that will be beneficial in the discovery of novel therapeutic biomarkers of PPBC.

## Introduction

Breast cancer is one of the leading causes of death of women globally as a result of metastatic disease [1]. Over the years, there has been a trend in women that have deferred to having their first child after the age of 35, therefore, increasing their risk of breast cancer development [2]. This subtype of breast cancer amongst postpartum women, termed postpartum breast cancer (PPBC), is diagnosed within 5–10 years following birth of the last child and is observed in women under 45 years of age [2–6]. With this definition, PPBC can be attributed to approximately half of the young mothers diagnosed with breast cancer during their postpartum period [2, 4, 6, 7]. PPBC tumors have been observed to be more advanced and generally have poor prognoses as a consequence of their distinct biology resulting from lactation and subsequent involution of the gland [2, 4, 8–10]. These processes ultimately create an inflammatory environment within the mammary gland [11]. An inflammatory response can be triggered by various internal or external influences such as pathogens, toxic compounds and cellular damage/tissue injury [12–15]. Inflammation resulting from non-pathogenic stimuli, such as cellular damage, is referred to as ‘sterile’ inflammation. Inflammation has been attributed to the pathogenesis of many diseases, including cancer [13, 16–18]; thus, it is widely accepted that inflammation is a hallmark of cancer development and is a major target in current immunotherapeutic treatments. The onset of inflammation triggers resolution cascades that exhibit wound healing attributes, inclusive of the infiltration of various immune cell types [14, 19]. Mammary gland involution is acknowledged to emulate a wound healing signature [20–23]; however, little effort has been made to confer the ‘wound’ that prompts this wound-healing environment. Here, we hope to open a dialogue into the consideration of lactation as the inflammatory ‘wound’ and involution as the wound healing-like modulator of the inflammatory response. The relationship between the inflammatory profile of the mammary gland and neoplastic development is an area of high interest; however, the mechanism of how post-pregnancy stages, specifically lactation, influence PPBC progression requires more rigorous evaluation. Specifically, we suggest that investigating immune pathway activation within differentiating mammary epithelial cells (MECs) will provide more comprehensive knowledge of how the immune system functionally contributes to mammary gland development, as it has been recently proposed that the effector functions of immune cells are partially mediated by MECs and the cytokines they produce [24].

In this review we aim to provide a broad overview of what is currently known of the role of immune cells during the stages of postnatal mammary gland development. We also highlight an area of interest for future studies regarding the exploration of innate immune activation within MECs as a mechanism to uncover potential biomarkers of inflammation that may be beneficial in improving current therapeutic options for young women afflicted with PPBC. In this review we have focused on mouse and human model systems. Studies of postnatal mammary development and PPBC in human subjects are limited due to the sparsity of human breast tissue making mouse models a suitable alternative, as the overall structure of the immune system between the two species is remarkedly similar [2, 25]. However, it is important to note any significant species differences to prevent assumptions of human immunology that either do not occur or can’t be modeled in murine systems [25].

## Pubertal Development

The mammary gland is a unique organ that undergoes three stages of development: embryonic development that occurs in utero, as well as pubertal and reproductive development that occur postnatally. Complex interactions between epithelial cells and immune cells within the stroma play an important role in postnatal mammary gland development [20]. As MECs differentiate during pregnancy and lactation, these immune cells are exposed to rarely encountered milk proteins that challenge self-tolerance mechanisms [23]; therefore, postnatal development of the mammary gland occurs in an immune-competent microenvironment [20, 26]. There has been extensive research into how cells of the innate immune system play a role in virgin development. At birth, the mammary gland begins as an epithelial rudimentary tree comprised of multiple layers of luminal ‘body’ cells that surround the central lumen and corresponding basal cells [27–29]. Resident macrophages have been found to be in high abundance adjacent to this rudimentary structure at ~ 2 weeks of age [30]. In the mouse, postnatal mammary gland development commences with puberty at ~ 3–4 weeks of age [26, 31, 32]. This developmental stage is characterized by ductal elongation and invasion as a result of proliferation of cells throughout the terminal end buds (TEBs). Previous studies have demonstrated that macrophages are responsible for compiling collagen into long structured fibers around TEBs that serve to enhance ductal elongation [20]. More recent evidence has determined that macrophages contribute to ductal elongation in a signal transducer and activator of transcription 5 (STAT5)-dependent manner [33]. In mammary organoids, it has been established that tumor necrosis factor alpha (TNFα) produced by macrophages activates the PIK3-CDK1/cyclin B1 signaling supporting the activity of mammary stem cells [34]. Ductal macrophages have also been determined to respond to tissue damage. Specifically, a multiphoton laser was utilized to generate epithelial cell damage. Following laser damage, 3D subcellular resolution intravital microscopy revealed that ductal macrophages extended dendrites towards the affected areas to facilitate the formation of a secure epithelial-immune interaction [35]. Mast cells have also been determined to be necessary for accurate TEB and duct formation, likely via degranulation [20, 29, 36–38]. Eosinophils have also been revealed to be recruited to the stroma area near the head of the proliferating TEBs to facilitate bifurcation [20, 30, 39, 40]. This infiltration is a result of augmented expression of the chemokine eotaxin at ~ 5 weeks of age [20]. Eosinophil infiltration must be tightly regulated as increased numbers were demonstrated to delay ductal growth [41]. Interestingly, decreased numbers of eosinophils resulted in diminished density in mammary gland structures [42, 43]. Taken together, this data is indicative that a delicate balance of immune cell quantity is required for normal development.

During pubertal development, myoepithelial cells begin laying down extracellular proteins including fibronectin, laminin and type IV collagen that will develop into the basement membrane, which separates the parenchymal and stromal regions [32]. The stromal compartment of the mammary gland surrounds the ducts and consists of thick connective tissue, fibroblasts as well as other cell types. Maintenance of ductal structures is contingent upon the health and strength of the basement membrane [31, 32]. Throughout differentiation, the basement membrane functions to influence epithelial characteristics including shape, growth, polarity and responsiveness to hormones [31, 32]; thus, the basement membrane is vital for MEC differentiation and overall function of the mammary gland. At 8–9 weeks of age, the ductal tree fills the entire fat pad [26, 44–46]. During each estrous cycle, the mammary gland expands in preparation for pregnancy, a time during which alveolar cells commence further differentiation into milk-secreting structures [26, 47]. If pregnancy does not occur, the gland regresses until the next estrous cycle, which occurs every 4–5 days in mice [29, 46, 48, 49].

Despite the contribution of innate immune cells during virgin development, there is little information regarding adaptive immune cell function throughout mammary gland development [37, 39, 40, 49]. A seminal paper from Plaks et al. was the first to shed light on how cells of the adaptive immune response functionally contribute to pubertal development [26]. In this study, they observed CD11c^+^ antigen presenting cells (APCs) in close proximity to the mammary epithelium during branching morphogenesis. Their results demonstrated that APCs actively interacted with and proliferated during organoid branching [26]. Based on these results, it was hypothesized that T cells would also need to interact closely with the epithelium due to APCs being necessary for proper T cell activation [26]. Indeed, they demonstrated that both CD4^+^ and CD8^+^ T cells were closely associated within mammary ducts and also co-localized with adjacent APCs [26]. A more recent study has identified that *CD11c-*Cre-mediated depletion of CD11b^−^CD103^+^ (DC1) dendritic cells (DCs) in immune regulatory factor 8(*IRF8*) floxed mice resulted in a small increase in branching within pubertal female mice compared to their control counterparts [35].

The most significant knowledge gained from the work from Plaks et al. came from the assessment of ductal growth in T cell receptor α (TCRα) deficient mice, which lack CD4^+^ and CD8^+^ T cells. It was found that there was accelerated ductal outgrowth as well as augmented branching in mammary glands lacking this T cell response [26]. These results were the first to demonstrate that CD4^+^ and CD8^+^ T cells negatively regulate mammary epithelial development. Additional studies will be required to have a more comprehensive understanding of various T cell subtypes throughout mammary gland development including pregnancy and lactation. Currently, data collected utilizing mouse models suggests that cells of innate and adaptive immunity exert contrasting effects during mammary gland development [50]. These results indicate that cells of the innate response serve to enhance pubertal development via secretion of cytokines including TNFα that stimulate proliferation of MECs. On the contrary, adaptive immune cells negatively regulate luminal differentiation via secretion of IFNγ [26, 50].

## Pregnancy

Pubertal mammary gland development is stimulated via hormonal signals from both the ovaries and pituitary gland; however, the pituitary hormone prolactin, is the most influential during pregnancy [44]. At the onset of pregnancy, upregulation of prolactin and progesterone promotes rapid proliferation of MECs, resulting in an increase in both epithelial cell number and surface area [44]. MECs also undergo a metabolic transition in which there are alterations to their gene expression profiles to favor impending demands for increases in energy production that are necessary for ensuing lactation [51, 52]. This period of augmented cell proliferation and metabolic activity generates replication fork stress as well as reactive oxygen species (ROS) that can promote oxidative damage to DNA, lipids and proteins that are required for cellular proliferation [53]. Replication stress is generally defined as a slowing or stalling of the replication fork complex [53, 54]; however, during pregnancy it is hypothesized to occur as a result of rapid proliferation of MECs [53]. During this time, epithelial cells are vulnerable to replication stress that may result in DNA damage if unrepaired. This damaged DNA has the potential to leak into the cytosol resulting in activation of cytosolic DNA sensors such as cyclic GMP-AMP synthase (cGAS). cGAS initiates activation of the stimulator of interferon genes (STING) that ultimately elicits downstream production of type I interferons and proinflammatory cytokines via activation of IRF3 and nuclear factor kappa B (NF-κB), respectively [55–58]. Studies have illustrated that throughout pregnancy, intracellular sensors will prompt activation of cellular stress responses such as those involved in preserving genome integrity, DNA damage repair, and cell cycle progression. Specifically, p53 and BRCA1 have been observed to be upregulated during pregnancy, to suppress tumor development and enhance DNA damage repair, respectively [53, 54, 59, 60]. There has also been emerging evidence suggesting crosstalk between pathways associated with genome stability and the immune response [15, 55, 61]. Specifically, innate immune mediators such as the cGAS-STING pathway have been demonstrated to be activated in response to DNA damage and chromosomal instability [62, 63]. Disruption of these protective mechanisms during pregnancy may predispose replicating MECs to unresolved DNA damage, further encouraging innate immune activation.

In response to hormonal cues at the onset of pregnancy, several regulatory networks are activated prompting alveolar cells to differentiate, resulting in the development of individual lobuloalveoli that are responsible for milk production and secretion during lactation [20, 30–32, 44, 47, 64, 65]. By the end of pregnancy, these lobuloalveolar structures will have replaced the adipocytes resulting in a functional milk-producing gland. Myoepithelial cells remain uniquely positioned around the alveoli to permit direct contact between epithelial cells and the basement membrane. This interaction is established to be essential for full MEC differentiation and subsequent milk secretion [32]. Due to their contractile nature, myoepithelial cells also serve to facilitate the movement of milk during lactation [31, 32]. Pregnancy denotes the most dramatic expansion of the mammary epithelium and alterations in the surrounding stroma. It has been previously reported that immune cells and their cytokines influence epithelial cell reorganization and differentiation into alveolar structures [20, 64, 66]; therefore, communication between epithelial and stromal cells is essential for differentiation into a milk-producing gland [22, 30, 31, 34, 40, 64, 67]. Studies regarding immune cell function in mammary gland development during pregnancy and lactation remain limited. During pregnancy, macrophages, eosinophils and mast cells have been established to accumulate adjacent to and in alignment with alveolar structures, though their functional contributions are less well-studied [20, 30, 38]. Despite this lack of knowledge, the infiltration of immune cells during pregnancy is hypothesized to contribute immune cells to the milk and effect immune surveillance due to increased risk of inflammation of the breast tissue that may involve infection, commonly referred to as mastitis [22, 68, 69]. There is some evidence suggestive that these cells also have important functional roles in development of the mammary gland during pregnancy. Mice lacking epithelial-associated macrophages exhibited pre-mature and aberrant alveoli formation during pregnancy in a colony stimulating factor 1 (CSF1)-dependent manner [29, 70]. This data is indicative that macrophages have important functional roles in mammary development, although more rigorous evaluation will be of benefit to further elucidate how other immune cells functionally contribute to pregnancy.

Throughout pregnancy, the mammary epithelium secretes cytokines to regulate lineage commitment and differentiation. In vitro, MECs in their undifferentiated state secrete type I cytokines such as IL12a, IFNγ and TNF [66, 71]. These cytokines favor subsequent development of a strong cellular immune response via stimulation and activation of both innate and adaptive immune cells such as natural killer (NK) cells, neutrophils, macrophages and T cells. Upon differentiation in the presence of lactogenic hormones such as prolactin and dexamethasone, MECs switch to production and secretion of type II cytokines such as IL4, IL13 and STAT6 [66, 71]. This type II response directs regulation of inflammation, antibody production as well as effector T cell responses. Transcriptome analysis of total RNA from mammary tissue during pregnancy and lactation exhibited increased expression of immune mediators such as T regulatory (T_reg_) cytokines [30]. T_regs_ secrete inhibitory cytokines such as TGFβ and IL10 to facilitate maintenance of cellular homeostasis and self-tolerance and may contribute to mammary gland development by mitigating the adaptive immune response, specifically, effector T cell functions. This data also illustrated augmented levels of IL4 and IL13 which are hypothesized to directly regulate MEC function in addition to influencing the surrounding immune cell population [71]. Interestingly, factors implicated in lineage commitment of luminal epithelial cells during pregnancy are known to also be associated with T cell lineage determination [71]. Specifically, a role of STAT6 and its upstream cytokines, IL4 and IL13, has previously been demonstrated in the expansion of the luminal lineage and is also established to reinforce commitment to a type 2 helper (T_H_2) T cell lineage [66, 71, 72]. Moreover, MECs in their undifferentiated state secrete type I cytokines, similarly to what has been observed in the polarization and commitment to the T_H_1 T cell lineage [71]. To date, there are few studies on leukocyte functional involvement during pregnancy and lactation aside from their roles in response to infection. Immune cells elicit vast phenotypic and compositional changes during mammary gland development [50]; thus, studies into the role of immune cells during pregnancy and lactation will be required to enhance our knowledge of leukocyte function under normal developmental conditions. Additionally, further investigations into the immune pathways responsible for the cytokine phenotype of differentiating MECs will delineate the mechanism(s) by which MECs communicate with surrounding immune cells to promote postnatal mammary development [67].

Prolactin is considered the main hormonal driver of mammary gland development during pregnancy and lactation. Several reports have demonstrated that prolactin can stimulate both innate and adaptive immune cells inclusive of natural killer (NK) cells, macrophages, dendritic cells (DCs), neutrophils, T cells and B cells [38, 65]. Recently, it has been hypothesized that prolactin plays a role in the homing of immune cells into the mammary gland, although the specific mechanism requires further exploration [38, 65]. Lymphocyte homing is a complex, controlled mechanism that is generally regulated via various factors such as cytokines, chemokines, and addressins [65]. It has been reported that prolactin signaling triggers expression of T cell chemokines such as chemokine (C-C motif) ligand 20 (CCL20) and chemokine (C-X-C motif) ligand 9 (CXCL9) in mammary epithelial cells [65, 73, 74]. These chemokines play a role in attracting lymphocytes and DCs and promoting differentiation and expansion of leukocytes to elicit tissue extravasation [65]; therefore, prolactin plays a role in indirect T cell mobilization from adjacent mucosa-associated lymphoid tissues to the mammary gland throughout pregnancy [38, 65]. This process likely involves multifaceted communication between the mammary epithelium and infiltrating T cells in addition to induction of chemokine and adhesion molecule expression. In support of this, recent studies have demonstrated that both macrophages and T cells are not only intimately associated with the mammary epithelium but are in fact embedded within the bilayer, which is ultimately suggestive of a necessity for epithelial-immune interactions [50, 67]. Interestingly, prolactin is also responsible for the movement of fluid, ions, and immune cells into the milk in preparation for lactation [38].

## Lactation

**Fig. 1:**
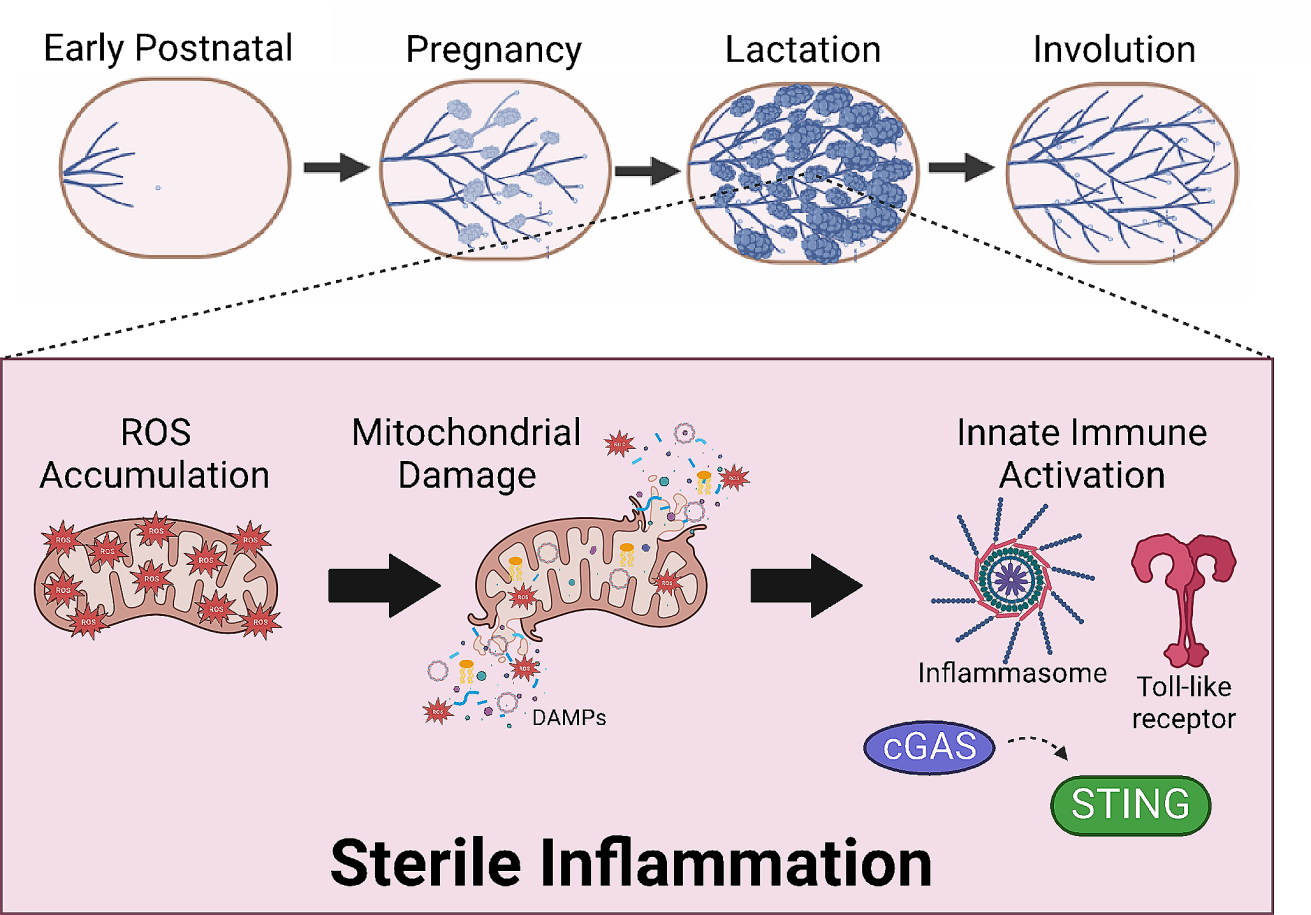
Lactation Challenges Mitochondrial Homeostasis. The lactating mammary gland challenges mitochondrial homeostasis via necessitating maintenance of a substantially demanding differentiated state. The mitochondria are under immense metabolic stress due to increased energetic demands as mammary epithelial cells work to produce milk. This stress results in augmented production of harmful reactive oxygen species (ROS) that result in mitochondrial damage/dysfunction. Under conditions of stress or damage, the mitochondria releases its components into the intracellular space to serve as damage associated molecular patterns (DAMPs). Aberrant mitochondrial components have been shown to elicit activation of innate immune modulators such as Toll-like receptors (TLRs), the inflammasome and the cGAS-STING pathway, all of which result in an inflammatory response. Ultimately, activation of innate immunity in response to mitochondrial damage promotes an environment of sterile inflammation; thus, innate immune activation during lactation is of interest for future studies

With the onset of lactation, there is a substantial increase in energetic demand as lobualveolar structures produce milk, which serves as a primary nutrient source for the offspring [51, 52]. During pregnancy, a metabolic shift commences in favor of supporting transport of glucose and amino acids to the developing fetus [75]. Once lactation begins, resource allocation adjusts from storage prioritization to augmented milk production in response to increased energy and nutrient demand [75]. Heightened energetic demand during lactation creates an enhanced probability of generating ROS which can cause cell damage. Reports have demonstrated that ROS can serve as signaling stimuli, however, a delicate balance between oxidants and antioxidants must be maintained to mitigate cellular damage [75]. MECs have been observed to upregulate several mechanisms to modulate cellular stress and damage triggered by lactogenic differentiation [52, 60, 76–79]. Mitochondrial function is vital to energy production; therefore, mitochondrial homeostasis must be maintained in metabolically active tissues such as the lactating mammary gland. Mitophagy, the process of recycling damaged mitochondria, is the primary way in which mitochondrial homeostasis is sustained [51, 52]. Moreover, this process is a key player in many mitochondrial stress responses. There is emerging evidence that supports a role of mitophagy during cellular differentiation as well as tissue development, a process that has been termed ‘programmed mitophagy’ [51, 80]. This process differs from other forms of nutrient or chemically induced mitophagy in that it occurs in response to developmental stimuli. Lactogenic differentiation results in severe metabolic stress [52] that results in damage to mitochondrial proteins, lipids and DNA, a consequence of which includes a loss of mitochondrial membrane integrity and potential [81–83]. Several lines of evidence suggest that mitochondria contribute to activation of innate immune pathways following cellular damage and stress due to the generation of ROS [84–86], and that maintenance of mitochondrial dynamics is vital in mammalian development [87]. ROS are key signaling molecules and also play a vital role in the progression of inflammatory diseases [84]. Release of mitochondrial components into the cytosol has been shown to result in activation of toll-like receptors (TLRs), the cGAS-STING pathway [87–89] and the inflammasome [90, 91], noting the intimate relationship between mitochondrial homeostasis and innate immune pathways [87–89, 92, 93]. Promoting mitophagy during MEC differentiation is required to gain more efficient mitochondria to meet the high energetic demands of lactation. Our lab has recently published that mitophagy is required for MEC differentiation [51]. Eliminating damaged mitochondria also aids in mitigating activation of innate immune mediators as MECs endure metabolic stress during lactation. These data suggest that lactation is likely a key driver of innate immune activation and highlights the need for further studies to assess innate immune activation within MECs (Fig. 1). Mitochondrial regulation of the immune system is relatively understudied in the mammary epithelium as past research of immunity during lactation have fixated on the transfer of passive immunity from the mother to the newborn during breastfeeding.

Breastfeeding promotes the postnatal transfer of nutrients and immune factors from the mother to the infant, enhancing protection against both respiratory and gastrointestinal infections in the immunologically naïve newborn [20, 94]. Immune cells passively transferred from mother to newborn serve to compensate for developmental delays in the infant immune system. This delay is thought to occur because, in utero, the fetus is protected by the maternal immune system from most pathogens and therefore does not require the mature immune system that is necessary for independent survival [94]; thus, nutritional resources and energy can be utilized for the development of essential organ systems that are vital for extrauterine survival [94]. Another reason for this delay may also be to circumvent immunologic reactions to maternal tissues in utero. Maternal milk consists of various nutrients, factors and cells that are beneficial upon ingestion by the newborn. Leukocytes are thought to migrate via lymphatic vessels and systemic circulation into the breast where it is believed they enter the lumen of the alveoli via the paracellular pathway [69]. Specifically, these immune cells must undergo a transepithelial migration in which they must cross the ductal basement membrane, make their way around the myoepithelial cells and finally transverse the luminal epithelial cell layer to enter the alveolar lumen [30, 38].

Previous studies have observed that macrophages and neutrophils are present in breast milk [Wirt 1992]. Breast milk leukocytes not only protect the infant but also protect the mother from infection during lactation. Conditions such as trauma, inadequate emptying of the breast, and ductal blockage can trigger an inflammatory response in the mammary gland, often resulting in severe mastitis, abscess or septicemia [69]. To facilitate in mitigation of these circumstances, there is an increased influx of leukocytes into the mammary gland. Functional alterations in breast milk leukocytes such as neutrophils and macrophages have been discovered in comparison to those present in the peripheral blood [94, 95]. Following stimulation with chemoattractants, it was observed that breast milk neutrophils and macrophages exhibited increased expression of CD11b as well as decreased expression of L-selectin [94]; therefore, the adherence, orientation and movement of breast milk neutrophils and macrophages were drastically diminished [95]. These leukocytes were also shown to exhibit known markers of activation [95]. Lymphocytes, inclusive of both B and T cells have also been observed in breast milk [38, 39, 69, 94, 95]. B cells are main components of adaptive humoral immunity. These cells serve to produce immunoglobulins, such as IgA, that enters the milk to elicit protection against intestinal and respiratory pathogens in the recipient infant [20, 94]. In support of this, IgA-producing B cells have been observed near alveolar cells so that IgA can be released into the milk [38, 39]. Moreover, IgG and IgM have also been found in breast milk, although in much smaller concentrations [94, 95]. Despite the importance of immunoglobulins in breast milk, most lymphocytes in breast milk are T cells, as determined by the presence of CD3 [94, 95]. It was verified that these T cells exhibited a memory phenotype as observed by expression of CD45RO as well as significantly more phenotypic markers of activation in comparison to T cells within the peripheral blood [69, 94, 95]. Breast milk T cells and macrophages illustrated a more motile phenotype compared to their blood counterparts, which is indicative of selective migration of these cells from the circulation into the milk [69]. Leukocytes within the milk have been observed to share morphologic characteristics similar to epithelial cells including the presence of lipid droplets, cytoplasmic vacuolation, as well as an enlarged nucleus or multinucleation [69]. Several pattern recognition receptors (PRRs), such as TLRs, that function to recognize molecular patterns of pathogens, have also been discovered in breast milk [96]. Interestingly, it has been hypothesized that lactation evolved as an inflammatory response to tissue damage and infection following birth, and that specific inflammatory molecules have become fundamental modulators of lactation [96]. We predict that exploration into immune activation within MECs will uncover inflammatory modulators of lactation. In support of this, past investigations have demonstrated that prolactin is not only a vital lactogenic hormone but can also serve as an anti-inflammatory or proinflammatory cytokine depending on the cellular milieu [96, 97]. This evidence implies that lactation exhibits a delicate inflammatory balance. Moreover, transcriptional single cell analysis using human milk suggests that essential cellular dynamics will enhance understanding of the association of lactation with breast cancer development [98].

During lactation, the mammary epithelium must become tolerogenic to milk components [22, 23, 99] and adopt a partially suppressive immune environment to permit tolerance to self-antigens [23, 100]. There is evidence that various immune cell types are present during lactation, although the mechanism(s) directing their functional contributions remain limited [29, 49, 101–103]. Work from Wilson et al. has demonstrated that chemokine receptors are responsible for directing macrophage dynamics during normal development [102]. On the other hand, a study from Cansever et al. demonstrated that mice deficient in lactation-induced macrophages did not exhibit alterations in milk protein content, or structural variations. This data is suggestive that macrophages are not essential to the foundation of tissue integrity within the lactating mammary gland [103] and may function to grant immune surveillance and enter the milk to facilitate passive immunity transfer to the newborn. Additional studies regarding the type and distribution of macrophage populations are reviewed elsewhere [101, 104]. Interestingly, progeny from IL5 deficient mice, that have diminished numbers of eosinophils, were observed to be underweight and also exhibited high preweaning mortality rates [42]. Taken together, this data is indicative that further investigations into all immune cell types will provide insight into their regulation and effector functions to provide a more comprehensive understanding of how the immune system influences lactation.

As described above, a proposed function of lactation-associated immune cells is to provide immune surveillance and protection against possible infection. Until recently, bacterial infection has been the alleged cause of mastitis; however, there is accumulating evidence that infection may be an indirect consequence of an unidentified primary cause of the disease [11, 105]. The World Health Organization recognizes milk stasis as an indirect origin of mastitis [106]. The build-up of milk within the breast increases intraductal pressure triggering sterile inflammation that may generally be followed by a secondary bacterial infection [11]. The theory that sterile inflammation could be a primary cause of mastitis is not a new concept [107], however, the scarcity of breast tissue from women with mastitis creates challenges in further investigations of sterile inflammation being a causal agent. It has also been illustrated that innate immune receptors recognize intra- and extracellular molecules such as aberrant DNA and cellular components released as a consequence of breast engorgement, ultimately resulting in a potent innate immune response and milk stasis [105, 108]. This evidence further supports that lactation has an inflammatory profile that is of interest for future studies regarding PPBC initiation.

Recently, work from Oakes et al. demonstrated that mutation in the oligoadenylate synthase 2 (OAS2), a sensor of viral dsRNA, resulted in postpartum failure of lactation in mice that exhibited normal mammary gland development [105]. They observed drastically diminished synthesis of milk proteins in addition to inhibition of proliferation, cell death and a robust interferon response. These results were also reiterated in vitro using HC11 and T47D mammary cells [105]. These studies established, for the first time, that stimulation of an innate immune pathway in MECs can elicit milk stasis, therefore, introducing a novel therapeutic target for anti-inflammatory therapy [105]. Further investigations of immune activation within MECs may facilitate our understanding of postpartum mammary gland development, ultimately improving postpartum breast health and decreasing instances of PPBC [67]. Lactation represents a time of metabolic stress that ultimately promotes sterile inflammation that challenges cellular homeostasis. During times of stress, cells either adapt or die [52]. Recent data has demonstrated decreased antigen-dependent T cell expansion during lactation that is predicted to assure successful lactation by increasing self-tolerance mechanisms to milk [23]. Taken together, this evidence maintains that there is a delicate balance of immune activation/suppression within the lactating mammary gland to encourage MEC survival during functional mammary gland development.

Current evidence leads us to anticipate that cancer cells utilize intrinsic mechanisms of immune suppression during lactation to evade immune surveillance and promote the development of a pro-tumorigenic microenvironment [109–112]. Increased expression of the inhibitory programmed death-ligand 1 (PDL1) in triple negative breast cancer reinforces this theory [113, 114]. Tumor cells express PDL1 which binds to its receptor, programmed death-1 (PD1), on T cells to inhibit their effector functions; thus, diminishing the adaptive immune response. Moreover, cancer cells have also demonstrated use of other normal cellular pathways such as those related to angiogenesis and sustained proliferation to facilitate their survival [115]. Several risk factors for cancer are associated with inflammatory mechanisms [13, 16–18], thus, the lactating mammary gland is becoming a conceivable environment to facilitate the promotion of PPBC development. Knowledge regarding the exact mechanisms of immune regulation during lactation remain limited; therefore, further exploration is required to elucidate how neoplastic cells utilize this time of inflammation to evade detection. We anticipate that investigations into this research area will uncover novel biomarkers for PPBC development, resulting in improved treatment options for women.

## Involution

Following lactation, the mammary gland undergoes involution resulting in a structure similar to that of the mature virgin gland [20]. Involution is categorized into two distinct phases: an acute, reversible phase followed by an irreversible phase. The acute phase is characterized by diminished synthesis and secretion of milk, in addition to STAT3-dependent lysosomal-mediated programmed cell death [29, 51, 116–118]. In the mouse, this initial phase typically occurs 1–3 days after weaning and is reversible. Generally, 72 h after weaning the irreversible phase begins. This phase is described as continued epithelial cell death, immune cell infiltration and tissue remodeling [21, 23, 35, 118–121]. This is followed by differentiation and repopulation of the stromal microenvironment by adipocytes [68]. CXCL1, a neutrophil chemoattractant, has been observed to be induced on day 1 of involution [20, 22]. By days 3–4, genes associated with macrophage chemoattraction, and differentiation were observed to be upregulated [22]. These include CCL6, CCL7, CCL8, and CXCL14 followed by macrophage-specific antigens such as CSF1 receptor (CSF1R), CD68 and low-density lipoprotein-related protein (LRP1), all of which exhibited peak expression 72–96 h post weaning [22]. In response to alterations in the surrounding stroma, monocytes mature into various macrophage phenotypes. During involution, monocytes specifically differentiate into M2 macrophages that are alternatively activated via cytokines such as IL4, IL10 and IL13 [20, 22]. This subtype of macrophages function in tissue remodeling, immunosuppression and wound repair [20]. Recent studies have uncovered that tissue resident macrophages are uniquely confined to the basal-luminal interface during involution as a superior position for surveillance of damaged and/or dying cells [120]. Ablation of mammary ductal tissue-resident macrophages achieved via either anti-CSF1R or *CD11c-DTR* (diphtheria toxin receptor) uncovered that other immune cell populations had diminished capabilities in the clearance of alveolar cells during involution [35]. Moreover, short-term depletion of CD11c^+^ cells with use of *CD11c-*DTR revealed delayed remodeling as determined by the persistence of sizeable alveolar lumens [35]. This was determined to be a result of a loss of CC3^+^ cell phagocytosis, noting the vital role of ductal macrophages in clearing cellular debris [119]. In addition to macrophages, eosinophils and mast cells have been observed to contribute to regeneration of adipocytes and ECM restructuring [40, 122, 123]. A unique subtype of NK T-cell like (NKT) cells have also recently been observed during late-stage involution [67]. These cells differ from typical NKT cells in that they express γδ T cell receptors (TCRs) instead of αβTCRs, indicating a role in specialized antigen recognition that permits inhibition of mammary oncogenesis [67]. Many of the infiltrating immune cells throughout mammary gland development are involved in wound healing [20]; therefore, enhancement of knowledge on the process of wound healing as well as the insulting agent triggering the inflammatory response will be beneficial for understanding immune cell function throughout postnatal mammary development.

Regulation of immune cell infiltration, activation and differentiation in the involuting mammary gland remains unknown. Previous data has suggested that involution is associated with an influx of neutrophils followed by macrophages and lymphocytes [68]. Effector T and B cells have been observed within the involuting mammary gland via the presence of CD4^+^, CD8^+^, and CD19^+^ cells, respectively [22]. These lymphocytes are largely believed to enhance defense against infection such as mastitis in the mother and respiratory and gastrointestinal infections in the newborn. Neutrophils are also critical in preventing mastitis as they function to eradicate bacteria through phagocytosis [68]. More recent studies are revealing that, in the mouse, epithelial cells are responsible for the clearance of most dead cells and residual milk proteins [20, 124]. Specifically, there has been evidence suggestive that MECs are capable of functioning as non-professional phagocytes to facilitate neutrophils and macrophages in the clearance of milk and cellular debris [20, 124]. In vitro, upon exposure to dead cells, MECs exhibited cytoskeletal actin reorganization, membrane ruffling and micropinocytosis [124]. Epithelial cells, endothelial cells and fibroblasts have been observed to function as amateur phagocytes to facilitate the removal of neighboring dead cells via phagocytosis [68]. Clearance of dead cells is crucial as it helps avert inflammation resulting from cell lysis through stimulating the secretion of anti-inflammatory cytokines [64, 71]. Dead cells that are not phagocytosed lose contact with the basement membrane and are released into the ductal lumen where they are accessible to professional phagocytes for disposal [68].

Mammary gland involution has been identified as a tumor promoting environment that contributes to the development and progression of PPBC [10, 22, 125]. Involution is known to exhibit wound healing inflammation and an immunosuppressive phenotype, both of which are hallmarks of cancer associated with tumor promotion and progression [23, 119, 120, 126–129]. This unique phenotype during involution likely occurs to support tolerance in a state of massive cell death. As previously described, STAT3 is an important mediator of programmed cell death within the involuting mammary gland. Importantly, STAT3 is also a key orchestrator of immune suppression [130] and has been revealed to be essential in promoting an immunosuppressive microenvironment when expressed in epithelial cells, ultimately, supporting tumor initiation and progression [131]. Additional mechanisms of how involution facilitates the development and progression of PPBC have been reviewed in further detail elsewhere [10, 22, 120, 125, 132–135].

## Conclusions

In conclusion, the mammary gland serves as a unique model to study how the immune system contributes to postnatal tissue development. Extensive work has been published on pubertal development and the process of involution, although mechanisms regarding immune cell functions that contribute to normal mammary development during pregnancy and lactation remain largely unexplored. In addition to the mammary gland, other tissues, including the brain and liver continue to develop postnatally. Emerging evidence has demonstrated that cytokines secreted by CD4^+^ T cells in the brain indirectly enhance spatial learning and memory as well as influence cell survival, proliferation and differentiation [136]. In the liver, a recent study demonstrated that signaling from macrophages resulted in recruitment of T_regs_ and thus, immunosuppressive activity during postnatal development [137]. In the breast, recent data is suggestive that pregnancy and lactation may permanently alter certain aspects of the molecular histology of the mammary gland, resulting in an increased risk for postpartum breast cancer development [4–6, 125, 138]. Involution is widely accepted as a driver of PPBC development due to its wound-healing attributes. Here, we suggest the consideration of lactation as a ‘wound’ that prompts the wound-healing signature during involution. Effector functions of immune cells within the developing mammary gland have been suggested to be influenced by MECs and they cytokines they produce. While functional contribution of immune cells is an area of high interest, we also propose that investigations into activation of innate immune pathways within MECs will be extremely beneficial in accessing how the immune system contributes to normal mammary development. We believe that future investigations aimed at accessing how lactation contributes to PPBC will facilitate the discovery of novel inflammatory biomarkers that can be targeted using immunotherapeutic treatment. Particularly, STING agonists have gained interest in breast cancer therapeutics and have shown promise in enhancing the efficacy of current immunotherapies [139–142]. Women diagnosed with PPBC have poorer prognoses and we believe that expanding our depth of knowledge regarding post-pregnancy developmental stages, particularly lactation, will facilitate the development for improved treatment options and better patient outcomes.

## Data Availability

No data was included in this manuscript.

## References

[1] Siegel RL, Miller KD, Wagle NS, Jemal A. Cancer statistics, 2023. CA Cancer J Clin. 2023;73(1):17–48. 10.3322/caac.21763. 36633525 10.3322/caac.21763

[2] Lefrere H, Lenaerts L, Borges VF, Schedin P, Neven P, Amant F. Postpartum breast cancer: mechanisms underlying its worse prognosis, treatment implications, and fertility preservation. Int J Gynecol Cancer. 2021;31(3):412–22. 10.1136/ijgc-2020-002072. 33649008 10.1136/ijgc-2020-002072 PMC7925817

[3] Lambe M, Hsieh C, Trichopoulos D, Ekbom A, Pavia M, Adami HO. Transient increase in the risk of breast cancer after giving birth. N Engl J Med. 1994;331(1):5–9. 10.1056/NEJM199407073310102. 8202106 10.1056/NEJM199407073310102

[4] Callihan EB, Gao D, Jindal S, Lyons TR, Manthey E, Edgerton S, Urquhart A, Schedin P, Borges VF. Postpartum diagnosis demonstrates a high risk for metastasis and merits an expanded definition of pregnancy-associated breast cancer. Breast Cancer Res Treat. 2013;138(2):549–59. 10.1007/s10549-013-2437-x. 23430224 10.1007/s10549-013-2437-x PMC3608871

[5] Faupel-Badger JM, Arcaro KF, Balkam JJ, Eliassen AH, Hassiotou F, Lebrilla CB, Michels KB, Palmer JR, Schedin P, Stuebe AM, Watson CJ, Sherman ME. Postpartum remodeling, lactation, and breast cancer risk: summary of a National Cancer Institute-sponsored workshop. J Natl Cancer Inst. 2013;105(3):166–74. 10.1093/jnci/djs505. 23264680 10.1093/jnci/djs505 PMC3611853

[6] Goddard ET, Bassale S, Schedin T, Jindal S, Johnston J, Cabral E, Latour E, Lyons TR, Mori M, Schedin PJ, Borges VF. Association between Postpartum breast Cancer diagnosis and metastasis and the clinical features underlying risk. JAMA Netw Open. 2019;2(1):e186997. 10.1001/jamanetworkopen.2018.6997. 30646210 10.1001/jamanetworkopen.2018.6997 PMC6484560

[7] Borges VF, Elder AM, Lyons TR. Deciphering pro-lymphangiogenic programs during mammary involution and postpartum breast Cancer. Front Oncol. 2016;6:227. 10.3389/fonc.2016.00227. 27853703 10.3389/fonc.2016.00227 PMC5090124

[8] Schedin P. Pregnancy-associated breast cancer and metastasis. Nat Rev Cancer. 2006;6(4):281–91. 10.1038/nrc1839. 16557280 10.1038/nrc1839

[9] Johansson AL, Andersson TM, Hsieh CC, Cnattingius S, Lambe M. Increased mortality in women with breast cancer detected during pregnancy and different periods postpartum. Cancer Epidemiol Biomarkers Prev. 2011;20(9):1865–72. 10.1158/1055-9965.EPI-11-0515. 21750168 10.1158/1055-9965.EPI-11-0515

[10] Wallace TR, Tarullo SE, Crump LS, Lyons TR. Studies of postpartum mammary gland involution reveal novel pro-metastatic mechanisms. J Cancer Metastasis Treat. 2019;5. 10.20517/2394-4722.2019.01. 10.20517/2394-4722.2019.01 PMC6400586 30847405

[11] Wockel A, Abou-Dakn M, Beggel A, Arck P. Inflammatory breast diseases during lactation: health effects on the newborn-a literature review. Mediators Inflamm. 2008;2008:298760. 10.1155/2008/298760. 18437232 10.1155/2008/298760 PMC2324165

[12] Medzhitov R. Inflammation 2010: new adventures of an old flame. Cell. 2010;140(6):771–6. 10.1016/j.cell.2010.03.006. 20303867 10.1016/j.cell.2010.03.006

[13] Nathan C, Ding A. Nonresolving inflammation. Cell. 2010;140(6):871–82. 10.1016/j.cell.2010.02.029. 20303877 10.1016/j.cell.2010.02.029

[14] Chen L, Deng H, Cui H, Fang J, Zuo Z, Deng J, Li Y, Wang X, Zhao L. Inflammatory responses and inflammation-associated diseases in organs. Oncotarget. 2018;9(6):7204–18. 10.18632/oncotarget.23208. 29467962 10.18632/oncotarget.23208 PMC5805548

[15] Nastasi C, Mannarino L, D’Incalci M. DNA damage response and Immune Defense. Int J Mol Sci. 2020;21(20). 10.3390/ijms21207504. 10.3390/ijms21207504 PMC7588887 33053746

[16] Dougan M, Dranoff G. Immune therapy for cancer. Annu Rev Immunol. 2009;27:83–117. 10.1146/annurev.immunol.021908.132544. 19007331 10.1146/annurev.immunol.021908.132544

[17] Zhao X, Xu Z, Li H. NSAIDs use and reduced metastasis in Cancer patients: results from a meta-analysis. Sci Rep. 2017;7(1):1875. 10.1038/s41598-017-01644-0. 28500305 10.1038/s41598-017-01644-0 PMC5431951

[18] Wong RSY. (2019). Role of Nonsteroidal Anti-Inflammatory Drugs (NSAIDs) in Cancer Prevention and Cancer Promotion. *Adv Pharmacol Sci*, *2019*, 3418975. 10.1155/2019/3418975. 10.1155/2019/3418975 PMC6374867 30838040

[19] Serhan CN, Savill J. Resolution of inflammation: the beginning programs the end. Nat Immunol. 2005;6(12):1191–7. 10.1038/ni1276. 16369558 10.1038/ni1276

[20] Reed JR, Schwertfeger KL. Immune cell location and function during post-natal mammary gland development. J Mammary Gland Biol Neoplasia. 2010;15(3):329–39. 10.1007/s10911-010-9188-7. 20730636 10.1007/s10911-010-9188-7 PMC4204476

[21] Scribner KC, Wellberg EA, Metz RP, Porter WW. Singleminded-2s (Sim2s) promotes delayed involution of the mouse mammary gland through suppression of Stat3 and NFkappaB. Mol Endocrinol. 2011;25(4):635–44. 10.1210/me.2010-0423. 21292822 10.1210/me.2010-0423 PMC3386548

[22] Fornetti J, Martinson HA, Betts CB, Lyons TR, Jindal S, Guo Q, Coussens LM, Borges VF, Schedin P. Mammary gland involution as an immunotherapeutic target for postpartum breast cancer. J Mammary Gland Biol Neoplasia. 2014;19(2):213–28. 10.1007/s10911-014-9322-z. 24952477 10.1007/s10911-014-9322-z PMC4363120

[23] Betts CB, Pennock ND, Caruso BP, Ruffell B, Borges VF, Schedin P. Mucosal immunity in the female murine mammary gland. J Immunol. 2018;201(2):734–46. 10.4049/jimmunol.1800023. 29884705 10.4049/jimmunol.1800023 PMC6036228

[24] Tower H, Dall G, Davey A, Stewart M, Lanteri P, Ruppert M, Lambouras M, Nasir I, Yeow S, Darcy PK, Ingman WV, Parker B, Haynes NM, Britt KL. Estrogen-induced immune changes within the normal mammary gland. Sci Rep. 2022;12(1):18986. 10.1038/s41598-022-21871-4. 36347875 10.1038/s41598-022-21871-4 PMC9643548

[25] Mestas J, Hughes CC. Of mice and not men: differences between mouse and human immunology. J Immunol. 2004;172(5):2731–8. 10.4049/jimmunol.172.5.2731. 14978070 10.4049/jimmunol.172.5.2731

[26] Plaks V, Boldajipour B, Linnemann JR, Nguyen NH, Kersten K, Wolf Y, Casbon AJ, Kong N, van den Bijgaart RJ, Sheppard D, Melton AC, Krummel MF, Werb Z. Adaptive Immune regulation of mammary postnatal organogenesis. Dev Cell. 2015;34(5):493–504. 10.1016/j.devcel.2015.07.015. 26321127 10.1016/j.devcel.2015.07.015 PMC4573906

[27] Williams JM, Daniel CW. Mammary ductal elongation: differentiation of myoepithelium and basal lamina during branching morphogenesis. Dev Biol. 1983;97(2):274–90. 10.1016/0012-1606(83)90086-6. 6852366 10.1016/0012-1606(83)90086-6

[28] Visvader JE. Keeping abreast of the mammary epithelial hierarchy and breast tumorigenesis. Genes Dev. 2009;23(22):2563–77. 10.1101/gad.1849509. 19933147 10.1101/gad.1849509 PMC2779757

[29] Dawson CA, Visvader JE. The Cellular Organization of the mammary gland: insights from Microscopy. J Mammary Gland Biol Neoplasia. 2021;26(1):71–85. 10.1007/s10911-021-09483-6. 33835387 10.1007/s10911-021-09483-6

[30] Coussens LM, Pollard JW. Leukocytes in mammary development and cancer. Cold Spring Harb Perspect Biol. 2011;3(3). 10.1101/cshperspect.a003285. 10.1101/cshperspect.a003285 PMC3039933 21123394

[31] Hennighausen L, Robinson GW. Think globally, act locally: the making of a mouse mammary gland. Genes Dev. 1998;12(4):449–55. 10.1101/gad.12.4.449. 9472013 10.1101/gad.12.4.449

[32] Richert MM, Schwertfeger KL, Ryder JW, Anderson SM. An atlas of mouse mammary gland development. J Mammary Gland Biol Neoplasia. 2000;5(2):227–41. 10.1023/a:1026499523505. 11149575 10.1023/a:1026499523505

[33] Brady NJ, Farrar MA, Schwertfeger KL. STAT5 deletion in macrophages alters ductal elongation and branching during mammary gland development. Dev Biol. 2017;428(1):232–44. 10.1016/j.ydbio.2017.06.007. 28606561 10.1016/j.ydbio.2017.06.007 PMC5621646

[34] Zhou Y, Ye Z, Wei W, Zhang M, Huang F, Li J, Cai C. Macrophages maintain mammary stem cell activity and mammary homeostasis via TNF-alpha-PI3K-Cdk1/Cyclin B1 axis. NPJ Regen Med. 2023;8(1):23. 10.1038/s41536-023-00296-1. 37130846 10.1038/s41536-023-00296-1 PMC10154328

[35] Dawson CA, Pal B, Vaillant F, Gandolfo LC, Liu Z, Bleriot C, Ginhoux F, Smyth GK, Lindeman GJ, Mueller SN, Rios AC, Visvader JE. Tissue-resident ductal macrophages survey the mammary epithelium and facilitate tissue remodelling. Nat Cell Biol. 2020;22(5):546–58. 10.1038/s41556-020-0505-0. 32341550 10.1038/s41556-020-0505-0

[36] Silberstein GB, Daniel CW. Glycosaminoglycans in the basal lamina and extracellular matrix of the developing mouse mammary duct. Dev Biol. 1982;90(1):215–22. 10.1016/0012-1606(82)90228-7. 6800862 10.1016/0012-1606(82)90228-7

[37] Lilla JN, Werb Z. Mast cells contribute to the stromal microenvironment in mammary gland branching morphogenesis. Dev Biol. 2010;337(1):124–33. 10.1016/j.ydbio.2009.10.021. 19850030 10.1016/j.ydbio.2009.10.021 PMC2787992

[38] Dill R, Walker AM. Role of Prolactin in Promotion of Immune Cell Migration into the mammary gland. J Mammary Gland Biol Neoplasia. 2017;22(1):13–26. 10.1007/s10911-016-9369-0. 27900586 10.1007/s10911-016-9369-0 PMC5313375

[39] Gouon-Evans V, Rothenberg ME, Pollard JW. Postnatal mammary gland development requires macrophages and eosinophils. Development. 2000;127(11):2269–82. 10.1242/dev.127.11.2269. 10804170 10.1242/dev.127.11.2269

[40] Gouon-Evans V, Lin EY, Pollard JW. Requirement of macrophages and eosinophils and their cytokines/chemokines for mammary gland development. Breast Cancer Res. 2002;4(4):155–64. 10.1186/bcr441. 12100741 10.1186/bcr441 PMC138736

[41] Sferruzzi-Perri AN, Robertson SA, Dent LA. Interleukin-5 transgene expression and eosinophilia are associated with retarded mammary gland development in mice. Biol Reprod. 2003;69(1):224–33. 10.1095/biolreprod.102.010611. 12620930 10.1095/biolreprod.102.010611

[42] Colbert DC, McGarry MP, O’Neill K, Lee NA, Lee JJ. Decreased size and survival of weanling mice in litters of IL-5-/ -mice are a consequence of the IL-5 deficiency in nursing dams. Contemp Top Lab Anim Sci. 2005;44(3):53–5. https://www.ncbi.nlm.nih.gov/pubmed/15934726. 15934726

[43] Gurtner A, Crepaz D, Arnold IC. Emerging functions of tissue-resident eosinophils. J Exp Med. 2023;220(7). 10.1084/jem.20221435. 10.1084/jem.20221435 PMC10276195 37326974

[44] Oakes SR, Rogers RL, Naylor MJ, Ormandy CJ. Prolactin regulation of mammary gland development. J Mammary Gland Biol Neoplasia. 2008;13(1):13–28. 10.1007/s10911-008-9069-5. 18219564 10.1007/s10911-008-9069-5

[45] Sternlicht MD. Key stages in mammary gland development: the cues that regulate ductal branching morphogenesis. Breast Cancer Res. 2006;8(1):201. 10.1186/bcr1368. 16524451 10.1186/bcr1368 PMC1413974

[46] Ramakrishnan R, Khan SA, Badve S. Morphological changes in breast tissue with menstrual cycle. Mod Pathol. 2002;15(12):1348–56. 10.1097/01.MP.0000039566.20817.46. 12481017 10.1097/01.MP.0000039566.20817.46

[47] Brisken C, Kaur S, Chavarria TE, Binart N, Sutherland RL, Weinberg RA, Kelly PA, Ormandy CJ. Prolactin controls mammary gland development via direct and indirect mechanisms. Dev Biol. 1999;210(1):96–106. 10.1006/dbio.1999.9271. 10364430 10.1006/dbio.1999.9271

[48] Macias H, Hinck L. Mammary gland development. Wiley Interdiscip Rev Dev Biol. 2012;1(4):533–57. 10.1002/wdev.35. 22844349 10.1002/wdev.35 PMC3404495

[49] Hitchcock JR, Hughes K, Harris OB, Watson CJ. Dynamic architectural interplay between leucocytes and mammary epithelial cells. FEBS J. 2020;287(2):250–66. 10.1111/febs.15126. 31691481 10.1111/febs.15126 PMC7003847

[50] Zirbes A, Joseph J, Lopez JC, Sayaman RW, Basam M, Seewaldt VL, LaBarge MA. Changes in Immune Cell types with age in breast are consistent with a decline in Immune Surveillance and increased immunosuppression. J Mammary Gland Biol Neoplasia. 2021;26(3):247–61. 10.1007/s10911-021-09495-2. 34341887 10.1007/s10911-021-09495-2 PMC8566425

[51] Elswood J, Pearson SJ, Payne HR, Barhoumi R, Rijnkels M,W, W. P. Autophagy regulates functional differentiation of mammary epithelial cells. Autophagy. 2021;17(2):420–38. 10.1080/15548627.2020.1720427. 31983267 10.1080/15548627.2020.1720427 PMC8007166

[52] Sanchez L, Epps J, Wall S, McQueen C, Pearson SJ, Scribner K, Wellberg EA, Giles ED, Rijnkels M, Porter WW. SIM2s directed parkin-mediated mitophagy promotes mammary epithelial cell differentiation. Cell Death Differ. 2023;30(6):1472–87. 10.1038/s41418-023-01146-9. 36966227 10.1038/s41418-023-01146-9 PMC10244402

[53] Xu X, Chen E, Mo L, Zhang L, Shao F, Miao K, Liu J, Su SM, Valecha M, Chan UI, Zheng H, Chen M, Chen W, Chen Q, Fu H, Aladjem MI, He Y, Deng CX. BRCA1 represses DNA replication initiation through antagonizing estrogen signaling and maintains genome stability in parallel with WEE1-MCM2 signaling during pregnancy. Hum Mol Genet. 2019;28(5):842–57. 10.1093/hmg/ddy398. 30445628 10.1093/hmg/ddy398 PMC6381318

[54] Pearson SJ, Elswood J, Barhoumi R, Ming-Whitfield B, Rijnkels M, Porter WW. Loss of SIM2s inhibits RAD51 binding and leads to unresolved replication stress. Breast Cancer Res. 2019;21(1):125. 10.1186/s13058-019-1207-z. 31775907 10.1186/s13058-019-1207-z PMC6882179

[55] Sato H, Jeggo PA, Shibata A. Regulation of programmed death-ligand 1 expression in response to DNA damage in cancer cells: implications for precision medicine. Cancer Sci. 2019;110(11):3415–23. 10.1111/cas.14197. 31513320 10.1111/cas.14197 PMC6824998

[56] Hopfner KP, Hornung V. Molecular mechanisms and cellular functions of cGAS-STING signalling. Nat Rev Mol Cell Biol. 2020;21(9):501–21. 10.1038/s41580-020-0244-x. 32424334 10.1038/s41580-020-0244-x

[57] Reislander T, Groelly FJ, Tarsounas M. DNA damage and Cancer immunotherapy: a STING in the Tale. Mol Cell. 2020;80(1):21–8. 10.1016/j.molcel.2020.07.026. 32810436 10.1016/j.molcel.2020.07.026

[58] Chen L, Cao SQ, Lin ZM, He SJ, Zuo JP. NOD-like receptors in autoimmune diseases. Acta Pharmacol Sin. 2021;42(11):1742–56. 10.1038/s41401-020-00603-2. 33589796 10.1038/s41401-020-00603-2 PMC8564530

[59] Johansson EM, Kannius-Janson M, Bjursell G, Nilsson J. The p53 tumor suppressor gene is regulated in vivo by nuclear factor 1-C2 in the mouse mammary gland during pregnancy. Oncogene. 2003;22(38):6061–70. 10.1038/sj.onc.1206884. 12955085 10.1038/sj.onc.1206884

[60] Avivar-Valderas A, Wen HC, Aguirre-Ghiso JA. Stress signaling and the shaping of the mammary tissue in development and cancer. Oncogene. 2014;33(48):5483–90. 10.1038/onc.2013.554. 24413078 10.1038/onc.2013.554 PMC4096615

[61] Mukherjee S, Abdisalaam S, Bhattacharya S, Srinivasan K, Sinha D, Asaithamby A. Mechanistic link between DNA damage sensing, repairing and signaling factors and immune signaling. Adv Protein Chem Struct Biol. 2019;115:297–324. 10.1016/bs.apcsb.2018.11.004. 30798935 10.1016/bs.apcsb.2018.11.004 PMC7043287

[62] Dunphy G, Flannery SM, Almine JF, Connolly DJ, Paulus C, Jonsson KL, Jakobsen MR, Nevels MM, Bowie AG, Unterholzner L. Non-canonical activation of the DNA sensing adaptor STING by ATM and IFI16 mediates NF-kappaB signaling after nuclear DNA damage. Mol Cell. 2018;71(5):745–e760745. 10.1016/j.molcel.2018.07.034. 30193098 10.1016/j.molcel.2018.07.034 PMC6127031

[63] Li J, Hubisz MJ, Earlie EM, Duran MA, Hong C, Varela AA, Lettera E, Deyell M, Tavora B, Havel JJ, Phyu SM, Amin AD, Budre K, Kamiya E, Cavallo JA, Garris C, Powell S, Reis-Filho JS, Wen H, Bakhoum SF. Non-cell-autonomous cancer progression from chromosomal instability. Nature. 2023;620(7976):1080–8. 10.1038/s41586-023-06464-z. 37612508 10.1038/s41586-023-06464-z PMC10468402

[64] Watson CJ, Oliver CH, Khaled WT. Cytokine signalling in mammary gland development. J Reprod Immunol. 2011;88(2):124–9. 10.1016/j.jri.2010.11.006. 21255846 10.1016/j.jri.2010.11.006

[65] Mackern-Oberti JP, Valdez SR, Vargas-Roig LM, Jahn GA. Impaired mammary gland T cell population during early lactation in hypoprolactinemic lactation-deficient rats. Reproduction. 2013;146(3):233–42. 10.1530/REP-12-0387. 23904563 10.1530/REP-12-0387

[66] Khaled WT, Read EK, Nicholson SE, Baxter FO, Brennan AJ, Came PJ, Sprigg N, McKenzie AN, Watson CJ. The IL-4/IL-13/Stat6 signalling pathway promotes luminal mammary epithelial cell development. Development. 2007;134(15):2739–50. 10.1242/dev.003194. 17611223 10.1242/dev.003194

[67] Hanasoge Somasundara AV, Moss MA, Feigman MJ, Chen C, Cyrill SL, Ciccone MF, Trousdell MC, Vollbrecht M, Li S, Kendall J, Beyaz S, Wilkinson JE, Dos Santos CO. Parity-induced changes to mammary epithelial cells control NKT cell expansion and mammary oncogenesis. Cell Rep. 2021;37(10):110099. 10.1016/j.celrep.2021.110099. 34879282 10.1016/j.celrep.2021.110099 PMC8719356

[68] Atabai K, Sheppard D, Werb Z. Roles of the innate immune system in mammary gland remodeling during involution. J Mammary Gland Biol Neoplasia. 2007;12(1):37–45. 10.1007/s10911-007-9036-6. 17286210 10.1007/s10911-007-9036-6 PMC2574498

[69] Hassiotou F, Geddes DT. Immune cell-mediated protection of the mammary gland and the infant during breastfeeding. Adv Nutr. 2015;6(3):267–75. 10.3945/an.114.007377. 25979492 10.3945/an.114.007377 PMC4424778

[70] Pollard JW, Hennighausen L. Colony stimulating factor 1 is required for mammary gland development during pregnancy. Proc Natl Acad Sci U S A. 1994;91(20):9312–6. 10.1073/pnas.91.20.9312. 7937762 10.1073/pnas.91.20.9312 PMC44802

[71] Watson CJ. Immune cell regulators in mouse mammary development and involution. J Anim Sci. 2009;87(13 Suppl):35–42. 10.2527/jas.2008-1333. 18849387 10.2527/jas.2008-1333

[72] Ansel KM, Djuretic I, Tanasa B, Rao A. Regulation of Th2 differentiation and Il4 locus accessibility. Annu Rev Immunol. 2006;24:607–56. 10.1146/annurev.immunol.23.021704.115821. 16551261 10.1146/annurev.immunol.23.021704.115821

[73] Kanda N, Watanabe S. Prolactin enhances interferon-gamma-induced production of CXC ligand 9 (CXCL9), CXCL10, and CXCL11 in human keratinocytes. Endocrinology. 2007;148(5):2317–25. 10.1210/en.2006-1639. 17255201 10.1210/en.2006-1639

[74] Kanda N, Shibata S, Tada Y, Nashiro K, Tamaki K, Watanabe S. Prolactin enhances basal and IL-17-induced CCL20 production by human keratinocytes. Eur J Immunol. 2009;39(4):996–1006. 10.1002/eji.200838852. 19350575 10.1002/eji.200838852

[75] Hyatt HW, Zhang Y, Hood WR, Kavazis AN. Lactation has persistent effects on a mother’s metabolism and mitochondrial function. Sci Rep. 2017;7(1):17118. 10.1038/s41598-017-17418-7. 29215072 10.1038/s41598-017-17418-7 PMC5719424

[76] Hadsell DL, Olea W, Wei J, Fiorotto ML, Matsunami RK, Engler DA, Collier RJ. Developmental regulation of mitochondrial biogenesis and function in the mouse mammary gland during a prolonged lactation cycle. Physiol Genomics. 2011;43(6):271–85. 10.1152/physiolgenomics.00133.2010. 21189371 10.1152/physiolgenomics.00133.2010

[77] Invernizzi G, Naeem A, Loor JJ. Short communication: endoplasmic reticulum stress gene network expression in bovine mammary tissue during the lactation cycle. J Dairy Sci. 2012;95(5):2562–6. 10.3168/jds.2011-4806. 22541483 10.3168/jds.2011-4806

[78] Shao Y, Zhao FQ. Emerging evidence of the physiological role of hypoxia in mammary development and lactation. J Anim Sci Biotechnol. 2014;5(1):9. 10.1186/2049-1891-5-9. 24444333 10.1186/2049-1891-5-9 PMC3929241

[79] Davis KR, Giesy SL, Long Q, Krumm CS, Harvatine KJ, Boisclair YR. XBP1 regulates the Biosynthetic Capacity of the Mammary Gland during Lactation by Controlling Epithelial Expansion and endoplasmic reticulum formation. Endocrinology. 2016;157(1):417–28. 10.1210/en.2015-1676. 26562262 10.1210/en.2015-1676

[80] Esteban-Martinez L, Sierra-Filardi E, McGreal RS, Salazar-Roa M, Marino G, Seco E, Durand S, Enot D, Grana O, Malumbres M, Cvekl A, Cuervo AM, Kroemer G, Boya P. Programmed mitophagy is essential for the glycolytic switch during cell differentiation. EMBO J. 2017;36(12):1688–706. 10.15252/embj.201695916. 28465321 10.15252/embj.201695916 PMC5470043

[81] Sukumar M, Liu J, Mehta GU, Patel SJ, Roychoudhuri R, Crompton JG, Klebanoff CA, Ji Y, Li P, Yu Z, Whitehill GD, Clever D, Eil RL, Palmer DC, Mitra S, Rao M, Keyvanfar K, Schrump DS, Wang E, Restifo NP. Mitochondrial membrane potential identifies cells with enhanced stemness for Cellular Therapy. Cell Metab. 2016;23(1):63–76. 10.1016/j.cmet.2015.11.002. 26674251 10.1016/j.cmet.2015.11.002 PMC4747432

[82] Mills EL, Kelly B, O’Neill LAJ. Mitochondria are the powerhouses of immunity. Nat Immunol. 2017;18(5):488–98. 10.1038/ni.3704. 28418387 10.1038/ni.3704

[83] Sandhir R, Halder A, Sunkaria A. Mitochondria as a centrally positioned hub in the innate immune response. Biochim Biophys Acta Mol Basis Dis. 2017;1863(5):1090–7. 10.1016/j.bbadis.2016.10.020. 27794419 10.1016/j.bbadis.2016.10.020

[84] Mittal M, Siddiqui MR, Tran K, Reddy SP, Malik AB. Reactive oxygen species in inflammation and tissue injury. Antioxid Redox Signal. 2014;20(7):1126–67. 10.1089/ars.2012.5149. 23991888 10.1089/ars.2012.5149 PMC3929010

[85] Nakahira K, Hisata S, Choi AM. The roles of mitochondrial damage-Associated molecular patterns in diseases. Antioxid Redox Signal. 2015;23(17):1329–50. 10.1089/ars.2015.6407. 26067258 10.1089/ars.2015.6407 PMC4685486

[86] Duhig K, Chappell LC, Shennan AH. Oxidative stress in pregnancy and reproduction. Obstet Med. 2016;9(3):113–6. 10.1177/1753495X16648495. 27630746 10.1177/1753495X16648495 PMC5010123

[87] West AP, Shadel GS, Ghosh S. Mitochondria in innate immune responses. Nat Rev Immunol. 2011;11(6):389–402. 10.1038/nri2975. 21597473 10.1038/nri2975 PMC4281487

[88] West AP, Khoury-Hanold W, Staron M, Tal MC, Pineda CM, Lang SM, Bestwick M, Duguay BA, Raimundo N, MacDuff DA, Kaech SM, Smiley JR, Means RE, Iwasaki A, Shadel GS. Mitochondrial DNA stress primes the antiviral innate immune response. Nature. 2015;520(7548):553–7. 10.1038/nature14156. 25642965 10.1038/nature14156 PMC4409480

[89] West AP, Shadel GS. Mitochondrial DNA in innate immune responses and inflammatory pathology. Nat Rev Immunol. 2017;17(6):363–75. 10.1038/nri.2017.21. 28393922 10.1038/nri.2017.21 PMC7289178

[90] Jin HS, Suh HW, Kim SJ, Jo EK. Mitochondrial Control of Innate Immunity and Inflammation. Immune Netw. 2017;17(2):77–88. 10.4110/in.2017.17.2.77. 28458619 10.4110/in.2017.17.2.77 PMC5407986

[91] West AP. Mitochondrial dysfunction as a trigger of innate immune responses and inflammation. Toxicology. 2017;391:54–63. 10.1016/j.tox.2017.07.016. 28765055 10.1016/j.tox.2017.07.016

[92] Riley JS, Tait SW. Mitochondrial DNA in inflammation and immunity. EMBO Rep. 2020;21(4):e49799. 10.15252/embr.201949799. 32202065 10.15252/embr.201949799 PMC7132203

[93] Fang C, Mo F, Liu L, Du J, Luo M, Men K, Na F, Wang W, Yang H, Wei X. Oxidized mitochondrial DNA sensing by STING signaling promotes the antitumor effect of an irradiated immunogenic cancer cell vaccine. Cell Mol Immunol. 2021;18(9):2211–23. 10.1038/s41423-020-0456-1. 32398808 10.1038/s41423-020-0456-1 PMC8429462

[94] Goldman AS, Chheda S, Garofalo R. Evolution of immunologic functions of the mammary gland and the postnatal development of immunity. Pediatr Res. 1998;43(2):155–62. 10.1203/00006450-199802000-00001. 9475278 10.1203/00006450-199802000-00001

[95] Wirt DP, Adkins LT, Palkowetz KH, Schmalstieg FC, Goldman AS. Activated and memory T lymphocytes in human milk. Cytometry. 1992;13(3):282–90. 10.1002/cyto.990130310. 1533582 10.1002/cyto.990130310

[96] Vorbach C, Capecchi MR, Penninger JM. Evolution of the mammary gland from the innate immune system? BioEssays. 2006;28(6):606–16. 10.1002/bies.20423. 16700061 10.1002/bies.20423

[97] Gagnerault MC, Touraine P, Savino W, Kelly PA, Dardenne M. Expression of prolactin receptors in murine lymphoid cells in normal and autoimmune situations. J Immunol. 1993;150(12):5673–81. https://www.ncbi.nlm.nih.gov/pubmed/8515082. 8515082 10.4049/jimmunol.150.12.5673

[98] Twigger AJ, Engelbrecht LK, Bach K, Schultz-Pernice I, Pensa S, Stenning J, Petricca S, Scheel CH, Khaled WT. Transcriptional changes in the mammary gland during lactation revealed by single cell sequencing of cells from human milk. Nat Commun. 2022;13(1):562. 10.1038/s41467-021-27895-0. 35091553 10.1038/s41467-021-27895-0 PMC8799659

[99] Ballard O, Morrow AL. Human milk composition: nutrients and bioactive factors. Pediatr Clin North Am. 2013;60(1):49–74. 10.1016/j.pcl.2012.10.002. 23178060 10.1016/j.pcl.2012.10.002 PMC3586783

[100] Nagy D, Gillis CMC, Davies K, Fowden AL, Rees P, Wills JW, Hughes K. Developing ovine mammary terminal duct lobular units have a dynamic mucosal and stromal immune microenvironment. Commun Biol. 2021;4(1):993. 10.1038/s42003-021-02502-6. 34417554 10.1038/s42003-021-02502-6 PMC8379191

[101] Hassel C, Gausseres B, Guzylack-Piriou L, Foucras G. Ductal macrophages Predominate in the Immune Landscape of the Lactating Mammary Gland. Front Immunol. 2021;12:754661. 10.3389/fimmu.2021.754661. 34745127 10.3389/fimmu.2021.754661 PMC8564477

[102] Wilson GJ, Fukuoka A, Vidler F, Graham GJ. Diverse myeloid cells are recruited to the developing and inflamed mammary gland. Immunology. 2022;165(2):206–18. 10.1111/imm.13430. 34775606 10.1111/imm.13430 PMC10357480

[103] Cansever D, Petrova E, Krishnarajah S, Mussak C, Welsh CA, Mildenberger W, Mulder K, Kreiner V, Roussel E, Stifter SA, Andreadou M, Zwicky P, Jurado NP, Rehrauer H, Tan G, Liu Z, Bleriot C, Ronchi F, Macpherson AJ, Greter M. Lactation-associated macrophages exist in murine mammary tissue and human milk. Nat Immunol. 2023;24(7):1098–109. 10.1038/s41590-023-01530-0. 37337103 10.1038/s41590-023-01530-0 PMC10307629

[104] Wilson GJ, Fukuoka A, Love SR, Kim J, Pingen M, Hayes AJ, Graham GJ. Chemokine receptors coordinately regulate macrophage dynamics and mammary gland development. Development. 2020;147(12). 10.1242/dev.187815. 10.1242/dev.187815 PMC7328164 32467242

[105] Oakes SR, Gallego-Ortega D, Stanford PM, Junankar S, Au WWY, Kikhtyak Z, von Korff A, Sergio CM, Law AMK, Castillo LE, Allerdice SL, Young AIJ, Piggin C, Whittle B, Bertram E, Naylor MJ, Roden DL, Donovan J, Korennykh A, Ormandy CJ. A mutation in the viral sensor 2’-5’-oligoadenylate synthetase 2 causes failure of lactation. PLoS Genet. 2017;13(11):e1007072. 10.1371/journal.pgen.1007072. 29117179 10.1371/journal.pgen.1007072 PMC5695588

[106] WHO. Mastitis: causes and management. WHO/FCH/CAH/0013; 2000.

[107] Fetherston C. Mastitis in lactating women: physiology or pathology? Breastfeed Rev. 2001;9(1):5–12. https://www.ncbi.nlm.nih.gov/pubmed/11424519. 11424519

[108] Ingman WV, Glynn DJ, Hutchinson MR. Inflammatory mediators in mastitis and lactation insufficiency. J Mammary Gland Biol Neoplasia. 2014;19(2):161–7. 10.1007/s10911-014-9325-9. 24961655 10.1007/s10911-014-9325-9

[109] Coussens LM, Werb Z. Inflammation and cancer. Nature. 2002;420(6917):860–7. 10.1038/nature01322. 12490959 10.1038/nature01322 PMC2803035

[110] Mantovani A, Allavena P, Sica A, Balkwill F. Cancer-related inflammation. Nature. 2008;454(7203):436–44. 10.1038/nature07205. 18650914 10.1038/nature07205

[111] Grivennikov SI, Greten FR, Karin M. Immunity, inflammation, and cancer. Cell. 2010;140(6):883–99. 10.1016/j.cell.2010.01.025. 20303878 10.1016/j.cell.2010.01.025 PMC2866629

[112] Saxena M, Yeretssian G. NOD-Like receptors: Master regulators of inflammation and Cancer. Front Immunol. 2014;5:327. 10.3389/fimmu.2014.00327. 25071785 10.3389/fimmu.2014.00327 PMC4095565

[113] Mittendorf EA, Philips AV, Meric-Bernstam F, Qiao N, Wu Y, Harrington S, Su X, Wang Y, Gonzalez-Angulo AM, Akcakanat A, Chawla A, Curran M, Hwu P, Sharma P, Litton JK, Molldrem JJ, Alatrash G. PD-L1 expression in triple-negative breast cancer. Cancer Immunol Res. 2014;2(4):361–70. 10.1158/2326-6066.CIR-13-0127. 24764583 10.1158/2326-6066.CIR-13-0127 PMC4000553

[114] Schutz F, Stefanovic S, Mayer L, von Au A, Domschke C, Sohn C. PD-1/PD-L1 pathway in breast Cancer. Oncol Res Treat. 2017;40(5):294–7. 10.1159/000464353. 28346916 10.1159/000464353

[115] Sever R, Brugge JS. Signal transduction in cancer. Cold Spring Harb Perspect Med. 2015;5(4). 10.1101/cshperspect.a006098. 10.1101/cshperspect.a006098 PMC4382731 25833940

[116] Kreuzaler PA, Staniszewska AD, Li W, Omidvar N, Kedjouar B, Turkson J, Poli V, Flavell RA, Clarkson RW, Watson CJ. Stat3 controls lysosomal-mediated cell death in vivo. Nat Cell Biol. 2011;13(3):303–9. 10.1038/ncb2171. 21336304 10.1038/ncb2171

[117] Watson CJ, Kreuzaler PA. Remodeling mechanisms of the mammary gland during involution. Int J Dev Biol. 2011;55(7–9):757–62. 10.1387/ijdb.113414cw. 22161832 10.1387/ijdb.113414cw

[118] Sargeant TJ, Lloyd-Lewis B, Resemann HK, Ramos-Montoya A, Skepper J, Watson CJ. Stat3 controls cell death during mammary gland involution by regulating uptake of milk fat globules and lysosomal membrane permeabilization. Nat Cell Biol. 2014;16(11):1057–68. 10.1038/ncb3043. 25283994 10.1038/ncb3043 PMC4216597

[119] Stein T, Morris JS, Davies CR, Weber-Hall SJ, Duffy MA, Heath VJ, Bell AK, Ferrier RK, Sandilands GP, Gusterson BA. Involution of the mouse mammary gland is associated with an immune cascade and an acute-phase response, involving LBP, CD14 and STAT3. Breast Cancer Res. 2004;6(2):R75–91. 10.1186/bcr753. 14979920 10.1186/bcr753 PMC400652

[120] Hitchcock J, Hughes K, Pensa S, Lloyd-Lewis B, Watson CJ. The immune environment of the mammary gland fluctuates during post-lactational regression and correlates with tumour growth rate. Development. 2022;149(8). 10.1242/dev.200162. 10.1242/dev.200162 PMC9124574 35420674

[121] Stein T, Salomonis N, Gusterson BA. Mammary gland involution as a multi-step process. J Mammary Gland Biol Neoplasia. 2007;12(1):25–35. 10.1007/s10911-007-9035-7. 17431797 10.1007/s10911-007-9035-7

[122] O’Brien J, Lyons T, Monks J, Lucia MS, Wilson RS, Hines L, Man YG, Borges V, Schedin P. Alternatively activated macrophages and collagen remodeling characterize the postpartum involuting mammary gland across species. Am J Pathol. 2010;176(3):1241–55. 10.2353/ajpath.2010.090735. 20110414 10.2353/ajpath.2010.090735 PMC2832146

[123] Ramirez RA, Lee A, Schedin P, Russell JS, Masso-Welch PA. Alterations in mast cell frequency and relationship to angiogenesis in the rat mammary gland during windows of physiologic tissue remodeling. Dev Dyn. 2012;241(5):890–900. 10.1002/dvdy.23778. 22431477 10.1002/dvdy.23778 PMC3979934

[124] Monks J, Smith-Steinhart C, Kruk ER, Fadok VA, Henson PM. Epithelial cells remove apoptotic epithelial cells during post-lactation involution of the mouse mammary gland. Biol Reprod. 2008;78(4):586–94. 10.1095/biolreprod.107.065045. 18057312 10.1095/biolreprod.107.065045

[125] Borges VF, Lyons TR, Germain D, Schedin P. Postpartum Involution and Cancer: an opportunity for targeted breast Cancer Prevention and treatments? Cancer Res. 2020;80(9):1790–8. 10.1158/0008-5472.CAN-19-3448. 32075799 10.1158/0008-5472.CAN-19-3448 PMC8285071

[126] Clarkson RW, Wayland MT, Lee J, Freeman T, Watson CJ. Gene expression profiling of mammary gland development reveals putative roles for death receptors and immune mediators in post-lactational regression. Breast Cancer Res. 2004;6(2):R92–109. 10.1186/bcr754. 14979921 10.1186/bcr754 PMC400653

[127] Schafer M, Werner S. Cancer as an overhealing wound: an old hypothesis revisited. Nat Rev Mol Cell Biol. 2008;9(8):628–38. 10.1038/nrm2455. 18628784 10.1038/nrm2455

[128] Hanahan D, Weinberg RA. Hallmarks of cancer: the next generation. Cell. 2011;144(5):646–74. 10.1016/j.cell.2011.02.013. 21376230 10.1016/j.cell.2011.02.013

[129] MacCarthy-Morrogh L, Martin P. The hallmarks of cancer are also the hallmarks of wound healing. Sci Signal. 2020;13(648). 10.1126/scisignal.aay8690. 10.1126/scisignal.aay8690 32900881

[130] Kritikou EA, Sharkey A, Abell K, Came PJ, Anderson E, Clarkson RW, Watson CJ. A dual, non-redundant, role for LIF as a regulator of development and STAT3-mediated cell death in mammary gland. Development. 2003;130(15):3459–68. 10.1242/dev.00578. 12810593 10.1242/dev.00578

[131] Jones LM, Broz ML, Ranger JJ, Ozcelik J, Ahn R, Zuo D, Ursini-Siegel J, Hallett MT, Krummel M, Muller WJ. STAT3 establishes an immunosuppressive microenvironment during the early stages of breast carcinogenesis to promote Tumor Growth and Metastasis. Cancer Res. 2016;76(6):1416–28. 10.1158/0008-5472.CAN-15-2770. 26719528 10.1158/0008-5472.CAN-15-2770 PMC5052827

[132] Lyons TR, O’Brien J, Borges VF, Conklin MW, Keely PJ, Eliceiri KW, Marusyk A, Tan AC, Schedin P. Postpartum mammary gland involution drives progression of ductal carcinoma in situ through collagen and COX-2. Nat Med. 2011;17(9):1109–15. 10.1038/nm.2416. 21822285 10.1038/nm.2416 PMC3888478

[133] Martinson HA, Jindal S, Durand-Rougely C, Borges VF, Schedin P. Wound healing-like immune program facilitates postpartum mammary gland involution and tumor progression. Int J Cancer. 2015;136(8):1803–13. 10.1002/ijc.29181. 25187059 10.1002/ijc.29181 PMC4324053

[134] Elder AM, Tamburini BAJ, Crump LS, Black SA, Wessells VM, Schedin PJ, Borges VF, Lyons TR. Semaphorin 7A promotes macrophage-mediated lymphatic remodeling during Postpartum Mammary Gland Involution and in breast Cancer. Cancer Res. 2018;78(22):6473–85. 10.1158/0008-5472.CAN-18-1642. 30254150 10.1158/0008-5472.CAN-18-1642 PMC6239927

[135] Tamburini BAJ, Elder AM, Finlon JM, Winter AB, Wessells VM, Borges VF, Lyons TR. PD-1 Blockade during Post-partum Involution reactivates the anti-tumor response and reduces lymphatic vessel density. Front Immunol. 2019;10:1313. 10.3389/fimmu.2019.01313. 31244852 10.3389/fimmu.2019.01313 PMC6579890

[136] Morimoto K, Nakajima K. Role of the Immune System in the development of the Central Nervous System. Front Neurosci. 2019;13:916. 10.3389/fnins.2019.00916. 31551681 10.3389/fnins.2019.00916 PMC6735264

[137] Liang Y, Kaneko K, Xin B, Lee J, Sun X, Zhang K, Feng GS. Temporal analyses of postnatal liver development and maturation by single-cell transcriptomics. Dev Cell. 2022;57(3):398–e414395. 10.1016/j.devcel.2022.01.004. 35134346 10.1016/j.devcel.2022.01.004 PMC8842999

[138] Slepicka PF, Cyrill SL, Dos Santos CO. Pregnancy and breast Cancer: pathways to Understand Risk and Prevention. Trends Mol Med. 2019;25(10):866–81. 10.1016/j.molmed.2019.06.003. 31383623 10.1016/j.molmed.2019.06.003

[139] Xu N, Palmer DC, Robeson AC, Shou P, Bommiasamy H, Laurie SJ, Willis C, Dotti G, Vincent BG, Restifo NP, Serody JS. STING agonist promotes CAR T cell trafficking and persistence in breast cancer. J Exp Med. 2021;218(2). 10.1084/jem.20200844. 10.1084/jem.20200844 PMC7780733 33382402

[140] Pantelidou C, Jadhav H, Kothari A, Liu R, Wulf GM, Guerriero JL, Shapiro GI. STING agonism enhances anti-tumor immune responses and therapeutic efficacy of PARP inhibition in BRCA-associated breast cancer. NPJ Breast Cancer. 2022;8(1):102. 10.1038/s41523-022-00471-5. 36068244 10.1038/s41523-022-00471-5 PMC9448789

[141] Yin M, Hu J, Yuan Z, Luo G, Yao J, Wang R, Liu D, Cao B, Wu W, Hu Z. STING agonist enhances the efficacy of programmed death-ligand 1 monoclonal antibody in breast cancer immunotherapy by activating the interferon-beta signalling pathway. Cell Cycle. 2022;21(8):767–79. 10.1080/15384101.2022.2029996. 35130108 10.1080/15384101.2022.2029996 PMC8973354

[142] Ying-Rui M, Bu-Fan B, Deng L, Rong S, Qian-Mei Z. Targeting the stimulator of interferon genes (STING) in breast cancer. Front Pharmacol. 2023;14:1199152. 10.3389/fphar.2023.1199152. 37448962 10.3389/fphar.2023.1199152 PMC10338072

